# Three drugs are unnecessary for treating paucibacillary leprosy—A critique of the WHO guidelines

**DOI:** 10.1371/journal.pntd.0007671

**Published:** 2019-10-31

**Authors:** Diana N. J. Lockwood, Saba Lambert, Aparna Srikantam, Joydeepa Darlong, V. V. Pai, C. Ruth Butlin, Barbara de Barros, Edessa Negera, Stephen L. Walker

**Affiliations:** 1 London School of Hygiene & Tropical Medicine, Faculty of Infectious Diseases, London, United Kingdom; 2 LEPRA-Blue Peter Public Health and Research Center, Hyderabad, India; 3 The Leprosy Mission Trust, New Delhi, India; 4 Bombay Leprosy Project, Mumbai, India; 5 The Leprosy Mission England and Wales, United Kingdom; Swiss Tropical and Public Health Institute, SWITZERLAND

New guidelines have been published by WHO on the diagnosis, treatment, and prevention of leprosy [1]. The recommendations include that all leprosy patients, paucibacillary (PB) and multibacillary (MB), be treated with three antibacterial drugs. We have examined the evidence used to make these recommendations and find it lacking.

The Global Leprosy Programme (GLP) is to be commended for recognising that the absence of evidence-based guidelines for leprosy is a barrier to meeting the challenges outlined in the Global Leprosy Strategy 2016–2020 [2]. The development of guidelines based on the Grading of Recommendations Assessment, Development and Evaluations (GRADE) methodology represents a modern approach to the synthesis of evidence to aid policy makers, programme managers, and healthcare workers.

The WHO leprosy guidelines were published after the Guidelines Development Group (GDG), an expert committee, considered the evidence generated by commissioned systematic reviews and formulated guidance. The External Review Group then reviewed the guidance for errors, clarity, and implications but did not review the GDG’s recommendations. Certain recommendations, although agreed upon unanimously by the GDG, are based on insufficient evidence.

Guidelines published by WHO carry an authority, which means they are likely to have considerable influence on policy decisions, and it is therefore essential that the recommendations are robust.

The WHO Steering Group (SG) developed key questions to be addressed by the guidelines on the role of diagnostic tests, antibacterial chemotherapy, preventive chemotherapy, and vaccines. We agree with the recommendation that no new tests are suitable for diagnosing patients with leprosy. It is very surprising that the SG did not devise any questions concerning leprosy reactions. Leprosy reactions, which are associated with nerve damage and disability, occur in more than 60% of patients with MB leprosy during the first 6 months of treatment [3, 4]. Reactions are difficult to manage and may require prolonged treatment with immunomodulatory agents. The absence of guidance for the effective and safe management of reactions is a major omission. The GLP had an informal consultation on treatment of reactions and prevention of disabilities in leprosy held in Chennai in December 2018 [5].

The guidelines give a conditional recommendation (for which the quality of evidence is rated as low) for changing the antibacterial regimen used to treat patients with PB leprosy. The guidelines recommend that PB leprosy continue to be treated for 6 months but with 3 drugs rather than 2. This harmonises the drug combinations used to treat PB and MB disease.

PB leprosy has been treated with rifampicin and dapsone since the introduction of WHO multidrug therapy (MDT) in 1982. The duration of treatment is 6 months. Rifampicin is taken monthly and dapsone daily. The risk of relapse for PB leprosy following PB MDT was investigated in 2 prospective studies in Malawi and Indonesia, which showed a relapse rate of 0.65/100 person-years at risk (PYAR) and 0.12/100 PYAR, respectively [6]. Relapse rates lower than 1.0/100 PYAR were felt to be acceptable [7]. We estimate that over 9 million patients have been treated with this combination.

We examined the evidence underlying the recommendation that PB patients be treated with rifampicin, dapsone, and clofazimine. The guidelines state, “For PB leprosy patients, there is evidence of better clinical outcomes with a 3-drug 6-month regimen compared with a 2-drug 6-month regimen.” The papers cited were clinical trials by Prasad and colleagues [8], Rao and colleagues [9], Butlin and colleagues [10], and Penna and colleagues [3]; a prospective study of adverse effects by Gonçalves and colleagues [11]; and a descriptive study by Hungria and colleagues [12] on the prediction of reactions. The studies by Butlin [10] and Penna [3] only reported results in MB patients and are not relevant to this recommendation.

Only the studies by Prasad [8] and Rao [9] examined the efficacy of 6 months of MB MDT compared with PB MDT for PB patients. The nonblinded, quasi-randomised study of 44 patients by Prasad and colleagues used “clinical improvement” as an outcome measure in patients receiving 6 months’ treatment with PB MDT or MB MDT. Clinical improvement was assessed using unvalidated criteria of uncertain reliability including reduction in size of skin lesion and decrease in nerve thickening. They report statistically significant improvement in these criteria at 6 months for those who received MB MDT. Prasad and colleagues reported that they did not “encounter side-effects of anti-leprosy drugs” but not how adverse effects were monitored. Rao and colleagues [9] performed an open comparative trial that enrolled 32 PB patients and graded clinical improvement as “good,” “moderate,” and “poor” based on modifications to an unvalidated scoring system. There were no significant differences between the PB patients who received PB MDT and those who received MB MDT for 6 months. Two of 18 (11.1%) PB patients developed clofazimine pigmentation. The evidence from these studies was deemed to be of very low quality with a high risk of bias in Annex 3.3 of the evidence summary.

The results of 2 other studies were examined by the GDG to make this recommendation. There was no significant difference in the frequency of leprosy reactions in 158 individuals with PB leprosy treated with 6 months’ PB MDT or 6 months’ MB MDT [12]. The study by Gonçalves and colleagues compared 20 patients taking PB MDT and 20 taking MB MDT for 6 months. There was a significant increase in the proportion of individuals with anaemia due to haemolysis in PB patients receiving MB MDT compared with those receiving PB MDT [11]. The prevalence of drug-induced anaemia was similar to that of MB patients. The authors discussed this as a possible adverse effect caused by an interaction between clofazimine and dapsone. Clofazimine caused pigmentation in 10% and xerosis in 30% of individuals with PB leprosy who received MB MDT. The 2 studies by Rao and Gonçalves report high rates of adverse effects in PB patients taking 3 drugs.

The 2-drug PB MDT regimen given for 6 months has been recommended for almost 40 years, and there is no evidence of it failing. It is surprising that the GDG recommended changes to the treatment of PB leprosy, particularly when this will affect children. There appears to be no clinical justification to alter a regimen given to millions of patients with great success. Based on the 2017 global leprosy figures, this new recommendation would have resulted in 40.1% (84,688) of leprosy patients receiving unnecessary clofazimine. A total of 16,979 new cases were detected in children in 2017 who are more likely to have PB leprosy and thus be affected by this change in treatment [13].

The addition of unnecessary clofazimine goes against an important principle of good medical practice that one treats patients with the minimum number of drugs. An ethical aspect of the recommendation is that it contravenes the principle of “Primum non nocerre,” or “Do no harm.” The guidelines state that “No study reported adverse events with clofazimine…,” which is not the case. The main adverse effect is skin pigmentation of both leprosy lesions and normal appearing skin. Increased skin pigmentation can be a distressing adverse effect of treatment and may have a negative impact on self-esteem and adherence to treatment. Clofazimine-induced skin pigmentation may disclose the diagnosis of leprosy to others, which may lead to stigmatization. Kumar and colleagues found that 9.8% of patients taking MB MDT stopped their medication because of clofazimine pigmentation, showing that it is a significant problem [14].

The focus group discussions conducted with individuals affected by leprosy, after the final meeting of the GDG, identified “a lack of proper explanation of the side-effects of skin discoloration due to clofazimine as a possible cause of interruption of treatment.” It is unclear what individuals who had been diagnosed with PB leprosy thought of clofazimine pigmentation, which they are unlikely to have experienced. The guidelines state that “in persons with PB leprosy who are very concerned about the potential skin discoloration due to clofazimine, an alternative regimen (i.e., 2-drug therapy) could be considered.” A lack of information regarding adverse effects of treatment was highlighted as a barrier to adherence by the participants of the focus groups. No advice is given about eliciting the concerns of PB patients about clofazimine prior to commencing MDT and facilitating an informed decision. The influence of these guidelines might cause healthcare workers to overstate the dubious benefits of clofazimine in patients with PB leprosy.

We recognise the attraction from a public health perspective of a single intervention to manage both PB and MB leprosy. The logistics of producing and distributing MDT would be simplified and potentially cheaper, and the guidelines argue that the impact of the misclassification of patients with MB disease as PB cases would be reduced, although the GDG rejected the use of 6 months’ MB MDT for all individuals diagnosed with MB leprosy. The guidelines state that misclassification of MB patients in the field “might be an issue” and that “…there is evidence of some degree of misclassification of PB based on lesion counts” but do not provide a citation for this assertion nor give an estimate of the size of the problem. In the International Federation of Anti-leprosy Associations (ILEP) Nerve Function Impairment and Reaction (INFIR) Cohort Study, 63.7% of new MB patients in India had negative slit-skin smears, which shows that actually PB patients are being included in the MB category rather than the other way round [15].

The guidelines do not provide a robust framework for monitoring any negative impact of clofazimine on the management of PB disease. Pharmacovigilance is recommended after the introduction of the 3-drug regime, but reliable data will be difficult to collect, and treatment completion rates may not be an accurate reflection of the stigmatizing effects of clofazimine.

It is surprising that monitoring for adverse effects of the other components of MDT is not mentioned. The adverse effects of dapsone are now recognised as a major cause of morbidity and mortality in leprosy patients. Dapsone Hypersensitivity Syndrome has a mortality of 10% [16]. Adverse effects need to be monitored, and a less toxic antibacterial regimen that does not contain dapsone would be preferable.

Far-reaching changes that will worsen leprosy treatment have been made based on insufficient evidence. The impressive and clinically relevant number of patients successfully treated with this regimen has not been considered during the evidence assessment.

We urge WHO to rethink these recommendations. We urge all national programme managers to be cautious in adopting the new PB regimen because it may worsen outcomes and does not have a sound scientific base. We urge WHO to give leadership on a research programme to answer these important questions. It would have been better to set up studies comparing treatment of PB patients with the 2 regimens to assess outcomes, adverse effects, and acceptability to patients.
